# Machine Learning‐Based Radiomics in Malignancy Prediction of Pancreatic Cystic Lesions: Evidence from Cyst Fluid Multi‐Omics

**DOI:** 10.1002/advs.202409488

**Published:** 2025-04-28

**Authors:** Sihang Cheng, Ge Hu, Shenbo Zhang, Rui Lv, Limeng Sun, Zhe Zhang, Zhengyu Jin, Yanyan Wu, Chen Huang, Lu Ye, Yunlu Feng, Zhe‐Sheng Chen, Zhiwei Wang, Huadan Xue, Aiming Yang

**Affiliations:** ^1^ Department of Radiology Peking Union Medical College Hospital Chinese Academy of Medical Sciences Beijing 100730 China; ^2^ Theranostics and Translational Research Center National Infrastructures for Translational Medicine Institute of Clinical Medicine Peking Union Medical College Hospital Chinese Academy of Medical Sciences and Peking Union Medical College Beijing 100730 China; ^3^ Department of Gastroenterology Peking Union Medical College Hospital Chinese Academy of Medical Sciences Beijing 100730 China; ^4^ Department of Interventional Radiology The Affiliated Panyu Central Hospital of Guangzhou Medical University Guangzhou 511400 China; ^5^ Interventional Center Chengdu First People's Hospital Chengdu 610041 China; ^6^ Department of Pharmaceutical Sciences College of Pharmacy and Health Sciences St. John's University Queens NY 11439 USA

**Keywords:** artificial intelligence, lipidomic, pancreatic cystic lesions, proteomic, radiomics

## Abstract

The malignant potential of pancreatic cystic lesions (PCLs) varies dramatically, leading to difficulties when making clinical decisions. This study aimed to develop noninvasive clinical‐radiomic models using preoperative CT images to predict the malignant potential of PCLs. It also investigates the biological mechanisms underlying these models. Patients from two retrospective and one prospective cohort, all undergoing surgical resection for PCLs, are divided into four datasets: training, internal test, external test, and prospective application sets. Eleven machine learning classifiers are employed to construct radiomic models based on selected features. Cyst fluid from the prospective cohort is collected for proteomic and lipidomic analysis. The radiomic models demonstrated high accuracy, with area under the receiver operating characteristic curves (AUCs) > 0.93 across the training (n = 262), internal test (n = 50), and external test (n = 50) sets. AUCs ranged from 0.92 to 0.96 for the prospective cohort (n = 34). Meanwhile, differentially‐expressed proteins and lipid molecules, along with their associated signaling pathways, are identified between high and low groups of clinical‐radiomic scores. This models can effectively and accurately predict the malignant potential of PCLs, with multi‐omics evidence suggesting the biological mechanisms involving secretion function and lipid metabolism underlying clinical‐radiomic models.

## Graphical abstract

This study develops noninvasive clinical‐radiomic models using preoperative CT images to predict the malignant potential of pancreatic cystic lesions. It demonstrates the models' high accuracy across various datasets, with AUCs exceeding 0.92. Additionally, multi‐omics analyses uncover key biological mechanisms, including secretion function and lipid metabolism, driving these predictive models.

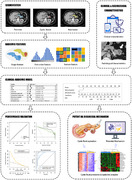

## 1 Introduction

Pancreatic cystic lesions (PCLs) have been increasingly detected in recent years with the rapid development of cross‐sectional imaging technologies.^[^ ^1^ ^]^ As a group of lesions with high heterogeneity, the malignant potential of PCLs varies between different types.^[^ ^2^ ^]^ Compared with serous cystic neoplasms (SCN), mucinous cysts, such as intraductal papillary mucinous neoplasms (IPMN) and mucinous cystic neoplasms (MCN), often show more aggressive biological behaviors, and a small subset of them may be precursors to the development of pancreatic ductal adenocarcinoma (PDAC), leading to poor prognosis.^[^ ^3^ ^]^ Thus, active surveillance and surgical resection are required to prevent malignant transformation of PCLs.^[^ ^4^ ^]^ With the accumulation of patients undergoing surveillance, medical costs and the use of healthcare resources increase dramatically. In addition, even in centers with high volume, the perioperative mortality for major pancreatic surgery ranges from 2%–4%.^[^ ^4^ ^]^ Therefore, accurate and cost‐effective diagnostic tools are urgently needed to differentiate PCLs with malignant potential from indolent cysts.

It is reported that PCLs are detected at a rate of 8% of all imaging examinations, with CT and MRI accounted for 2.7% and 24.8%, respectively.^[^ ^5^ ^]^ Although MRI performs better in cysts detection, CT is more widely available and time‐consuming with higher spatial resolution.^[^ ^1^ ^]^ Unfortunately, the diagnostic accuracy for the identification of a specific type of PCL is between 40% and 81% for CT, which is relatively low.^[^ ^6^ ^]^ Several imaging features, including worrisome features and high‐risk stigmata, are recommended by five major guidelines for the implication of malignant potential.^[^ ^6^, ^7^, ^8^, ^9^ ^]^ Notably, mural nodules of ≥ 5 mm and contrast‐enhanced solid components are characterized as high risk in all guidelines and found to be closely associated with high‐grade dysplasia or invasive IPMN.^[^ ^10^ ^]^ A machine learning model based on selected worrisome and high‐risk radiological features achieves high accuracy in identifying malignant PCLs.^[^ ^11^ ^]^ Nevertheless, the evaluation of imaging features differs between radiologists, leading to interobserver variants inevitably.

Radiomics has been used as a noninvasive tool for the quantitative investigation of radiological images. This data‐driven analysis is based on the hypothesis that the underlying pathophysiological characteristics of the imaged region could be reflected by a substantial amount of imaging features, also called radiomic biomarkers, extracted from medical images using high‐throughput techniques.^[^ ^12^ ^]^ The grey‐level co‐occurrence matrix (GLCM) quantifies the joint probability of pixel pairs exhibiting specific grey‐level values and has been shown to serve as a biomarker for treatment response in lung cancer patients undergoing immunotherapy, due to its sensitivity to hypoxia.^[^ ^13^ ^]^ Entropy, which measures the randomness of intensity in an image, has been linked to immune response through various molecular pathways, influencing macroscopic tumor changes that affect image textures.^[^ ^14^ ^]^ Tomaszewski and Gillies have summarized a series of radiomic biomarkers with their associated biological implications.^[^ ^15^ ^]^ Various studies have been conducted to stratify PCLs with the help of radiomics, and the results showed satisfactory performance of these radiomic models in classification of subtypes and assessment of malignant potential.^[^ ^16^, ^17^, ^18^, ^19^ ^]^ However, most previous studies were retrospectively designed based on relatively small sample sizes, without external or prospective validation performed independently. Furthermore, the biological underpinning of radiomic features associated with malignant potential of PCLs has not been elucidated, while increasing evidence has demonstrated the biological meaning of specific radiomic patterns in other tumor types.^[^ ^12^ ^]^ Thus, further studies focusing on the radiomic features of malignancy and related biological mechanisms should be carried out to guide clinical practice.

In this study, we aimed to develop a radiomic model for the prediction of PCLs with malignant potential based on preoperative CT images from a retrospective cohort and validate this model in independent cohorts externally and prospectively. Besides, cyst fluid from the prospective cohort was collected for proteomic and lipidomic analysis, trying to reveal the expression patterns of proteins and lipids in PCLs patients with high‐risk clinical‐radiomic features. In this way, the biological mechanism behind the models of malignancy prediction of PCLs was further explored.

## 2 Results

### 2.1 Patient Characteristics

**Figure 1:**
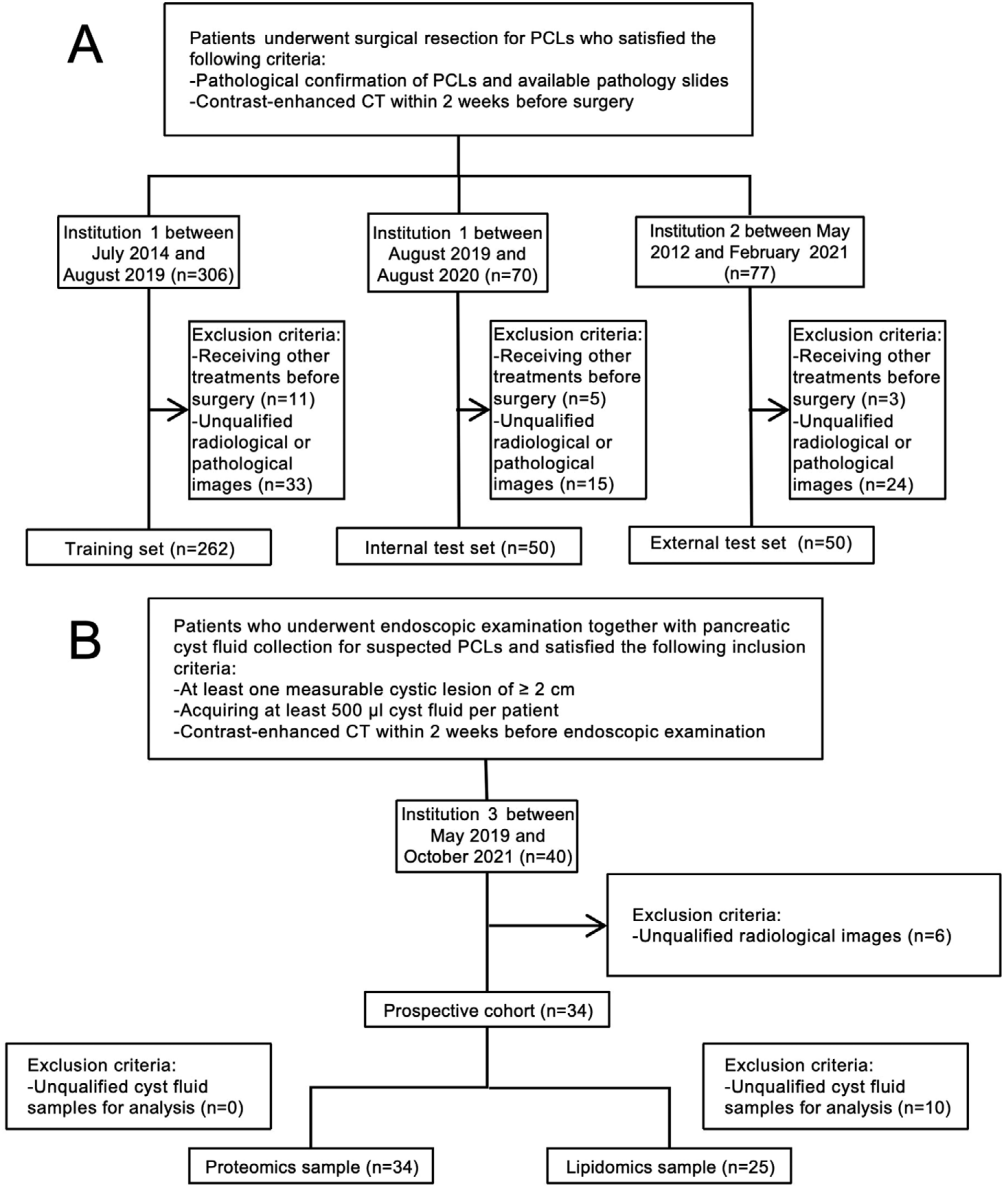
Flowcharts show patient recruitment process. A) Training set, internal test set, and external test set. B) Prospective cohort.

A total of 362 patients (150 men; 49 years ± 15) were finally included in the retrospective cohorts (**Figure** **1**A). The clinical characteristics and conventional image features of the study sample in the training set (262 patients from hospital 1 between July 2014 and August 2019) and internal (50 patients from hospital 1 between August 2019 and August 2020) and external (50 patients from hospital 2 between May 2012 and February 2021) test sets are summarized in Tables S1 and S2 (Supporting Information). All clinicopathologic characteristics and visual assessment features showed no difference between the three datasets (P > 0.05 for all).

In the training set (Tables S1 and S2, Supporting Information), 101 of 262 patients (39%) had malignant PCLs, and these patients were more likely to have high CEA (>5 ng/ml) (*P* < 0.001), thickened cystic wall (>2 mm; *P* < 0.001), enhanced cystic wall (*P* < 0.001), presence of septation (P = 0.008), thickened septation (>2 mm; *P* < 0.001), enhanced septation (P = 0.003), and solid component (*P* < 0.001). In the internal and external test sets, 40% (20 of 50) and 54% (27 of 50) patients, respectively, had malignant PCLs (Table S1, Supporting Information).

### 2.2 Generation of the Radiomic Models

**Figure 2:**
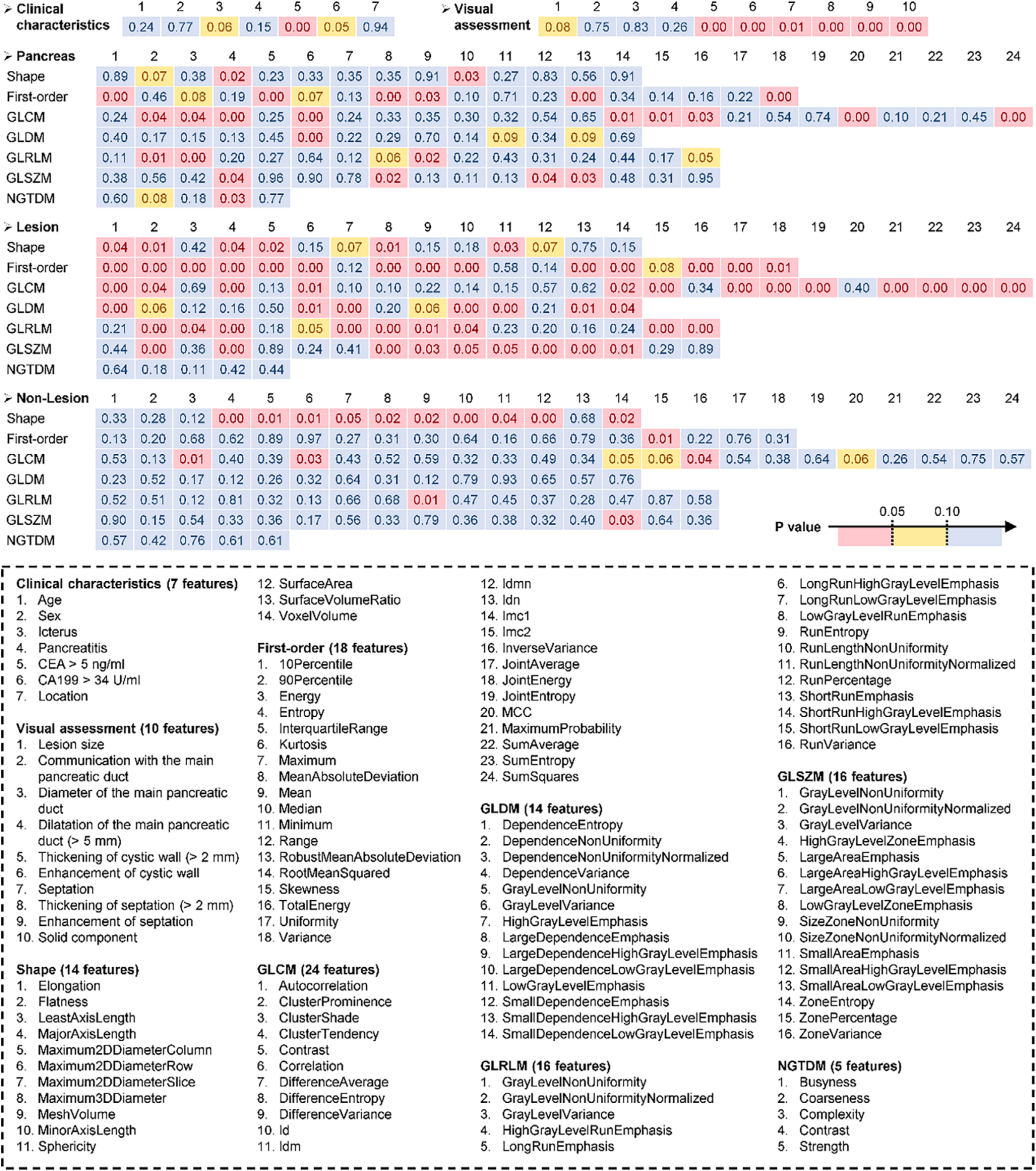
Univariable analysis of clinical characteristics, visual assessment features, and radiomic features in the training set. (Top) P values of clinical characteristics, visual assessment features, pancreas radiomic features, lesion radiomic features, and non‐lesion radiomic features. The values in red, yellow, and blue areas represent *p* < 0.05, 0.05 < *p* < 0.10, and *p* > 0.10, respectively. (Bottom) Feature index. The radiomic features contain 107 statistics in 7 categories: 14 3D shape features, 18 first‐order features, 24 GLCM features, 14 GLDM features, 16 GLRLM features, 16 GLSZM features, and 5 NGTDM features.

**Figure 3:**
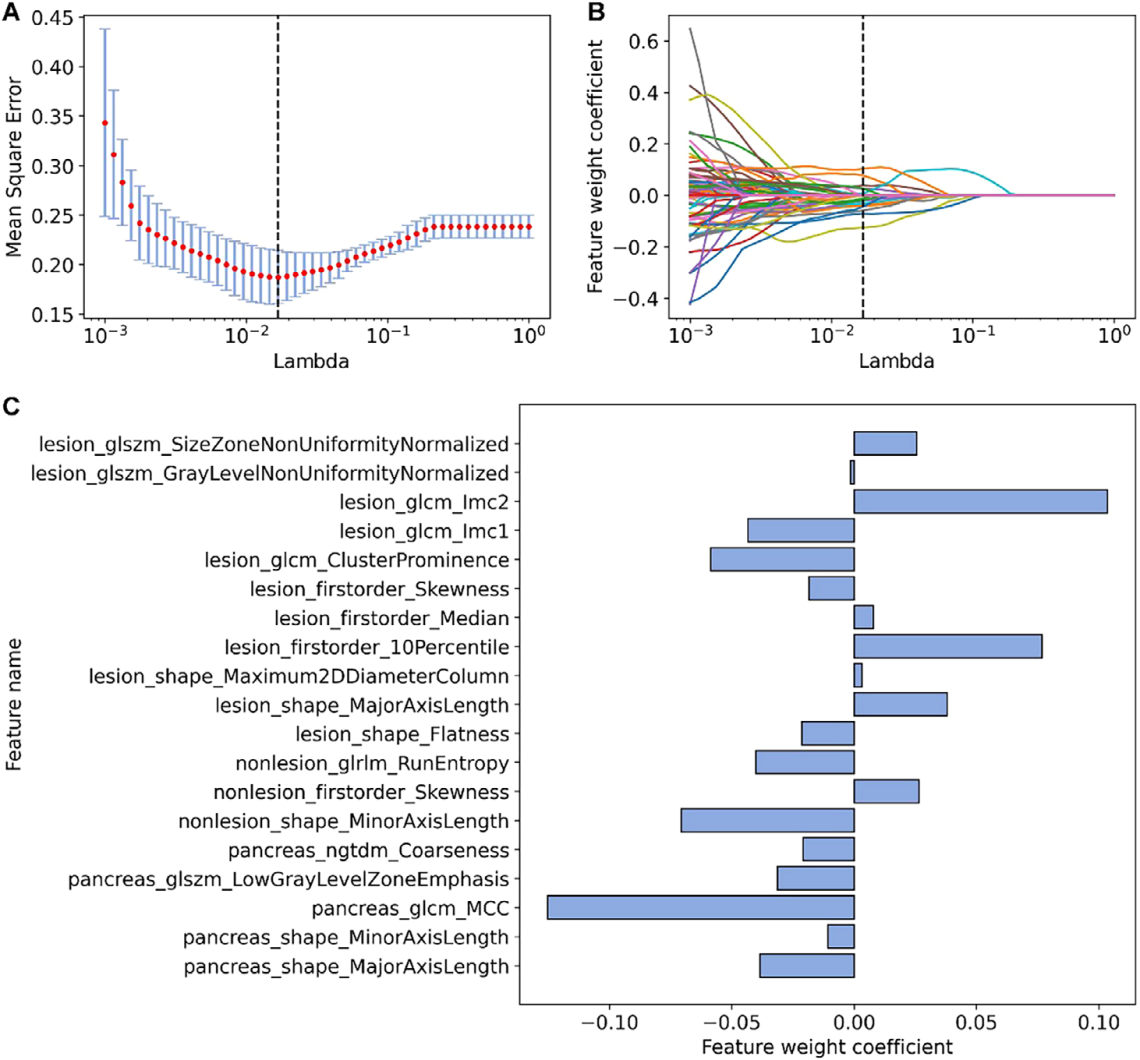
Multivariable selection of radiomic features based on LASSO regression. A) The trend graph of the mean square error (MSE) with different Lambda (λ) during cross‐validation. λ is an important parameter of LASSO regression that is usually adjusted by cross‐validation to find the optimal value. The red dots represent the average values of the MSE. The blue error bars represent the standard deviation of the MSE. The black dotted line indicates the best value of λ. B) The convergence graph of the weight coefficients of the features under different λ values. Each convergence line corresponds to a radiomic feature selected from the univariable analysis. As shown in (A) and (B), the MSE is minimized (0.2077 ± 0.0095) at λ = 0.0168 (the black dotted line), where 19 representative features were finally identified (weight coefficient ≠ 0). C) Feature names and weight coefficients of the 19 selected features.

We performed a univariable statistical analysis on the 321 radiomic features of the training set and retained 100 features with *P* < 0.05 and 17 features with *P* < 0.10 for subsequent least absolute shrinkage and selection operator (LASSO) feature selection (**Figure** **2**). Among these features, 55% (64 of 117) were lesion features, 29% (34 of 117) are pancreatic features, and 16% (19 of 117) are non‐lesion features. **Figure** **3** shows the results of LASSO regression. Figure 3A,B represents the change of the mean square error (MSE) of LASSO and the weight coefficients of the 117 features under different Lambda (λ) values, respectively. λ is an important parameter of LASSO regression that is usually adjusted by cross‐validation to find the optimal value. As shown in Figure 3A,B, the MSE is minimized (0.2077 ± 0.0095) at λ = 0.0168 (the black dotted line), where 19 radiomic features are finally identified (weight coefficient ≠ 0). Figure 3C shows the coefficients of the final selected features.

**Figure 4:**
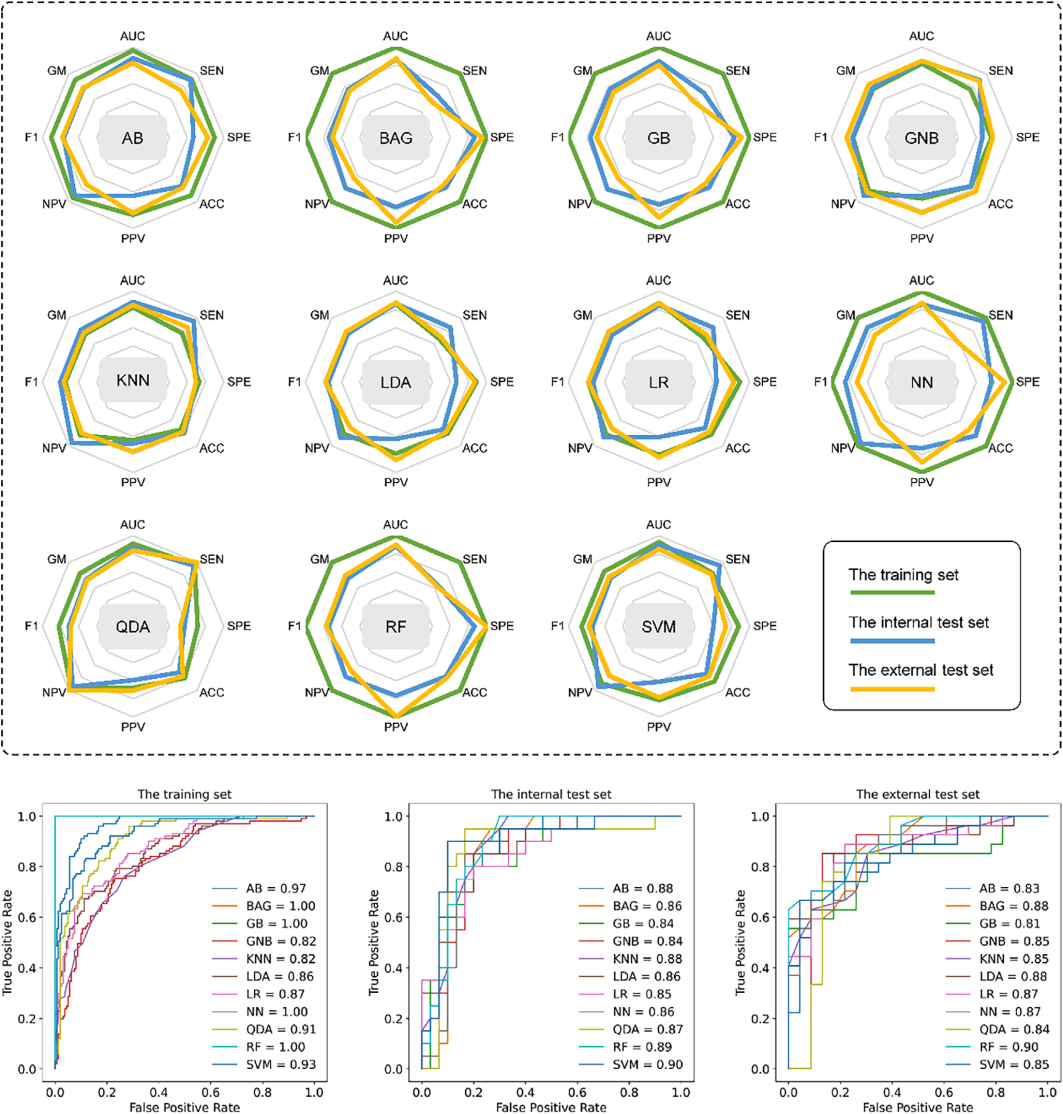
Performance of the machine learning models based on radiomic features. (Top) Radar maps of the eight performance indicators in different dataset for all eleven radiomic machine learning models. (Bottom) Performance of the radiomic models for predicting benign and malignant of pancreatic cystic neoplasm with receiver operating characteristic curve analysis in the training set, internal test set, and external test set.

Based on the selected radiomic features, we developed 11 radiomic models based on common machine learning classifiers. **Figure** **4** presents the radar maps of the eight evaluation indices and the ROC curves of the radiomic models on the training and test sets. All models had AUCs > 0.81 on the three datasets. Specifically, the highest AUC on the training set was 1.00 (BAG, GB, NN, RF) and the lowest AUC was 0.82 (GNB, KNN). On the internal test set, the highest and lowest AUC were 0.90 (SVM) and 0.84 (GB, GNB), respectively, and on the external test set were 0.90 (RF) and 0.81 (GB), respectively. Details of the performance indicators of the radiomic models can be found in Table S3 (training set), Table S4 (internal test set), and Table S5 (Supporting Information) (external test set). Figure S1 (Supporting Information) shows the radiomic model generated based on the AB radiomic score and its performance on different datasets.

### 2.3 Generation of the Clinical‐Radiomic Models

Based on univariable analysis results, clinical features with *P* < 0.10 (icterus, carcinoembryonic antigen (CEA), cancer antigen 199 (CA199), lesion size, cystic wall thickening, cystic wall enhancement, presence of septation, septation thickening, septation enhancement, and solid component) and the rescaled radiomic score were included in multivariable regression (Figure 2).

The results of the multivariate analyses were slightly different when different radiomic score were included, but all analysis results showed that the radiomic score and solid component still retained statistical significance in multivariable regression. Therefore, we generated 11 clinical‐radiomic models by combining the solid component and the different radiomic scores. The performance of the models is shown in **Table** **1**. All models had area under the receiver operating characteristic curves (AUCs) > 0.93 on the three datasets.

**Table 1:** Performance of the clinical‐radiomic models on different datasets.

Model	AUC of training set	AUC of internal test set	AUC of external test set
AB	0.98 [0.97, 0.99]	0.96 [0.90, 1.00]	0.96 [0.90, 1.00]
BAG	1.00 [1.00, 1.00]	0.94 [0.85, 1.00]	0.97 [0.92, 1.00]
GB	1.00 [1.00, 1.00]	0.93 [0.86, 1.00]	0.96 [0.90, 1.00]
GNB	0.93 [0.90, 0.96]	0.94 [0.86, 1.00]	0.97 [0.92, 1.00]
KNN	0.93 [0.90, 0.96]	0.94 [0.87, 1.00]	0.95 [0.88, 1.00]
LDA	0.94 [0.91, 0.97]	0.95 [0.88, 1.00]	0.96 [0.90, 1.00]
LR	0.94 [0.92, 0.97]	0.95 [0.88, 1.00]	0.96 [0.90, 1.00]
NN	1.00 [1.00, 1.00]	0.94 [0.85, 1.00]	0.97 [0.93, 1.00]
QDA	0.95 [0.93, 0.98]	0.96 [0.90, 1.00]	0.96 [0.92, 1.00]
RF	1.00 [1.00, 1.00]	0.95 [0.87, 1.00]	0.97 [0.93, 1.00]
SVM	0.96 [0.94, 0.98]	0.96 [0.90, 1.00]	0.96 [0.91, 1.00]

Data are AUCs with 95% confidence interval in square brackets. *Abbreviations*: AUC = area under the receiver operating characteristic curves, AB = adaptive boosting, BAG = bagging, GB = gradient boosting, GNB = Gaussian naive Bayes, KNN = k‐nearest neighbors, LDA = linear discriminant analysis, LR = logistic regression, NN = neural network, QDA = quadratic discriminant analysis, RF = random forest, SVM = support vector machine.

**Figure 5:**
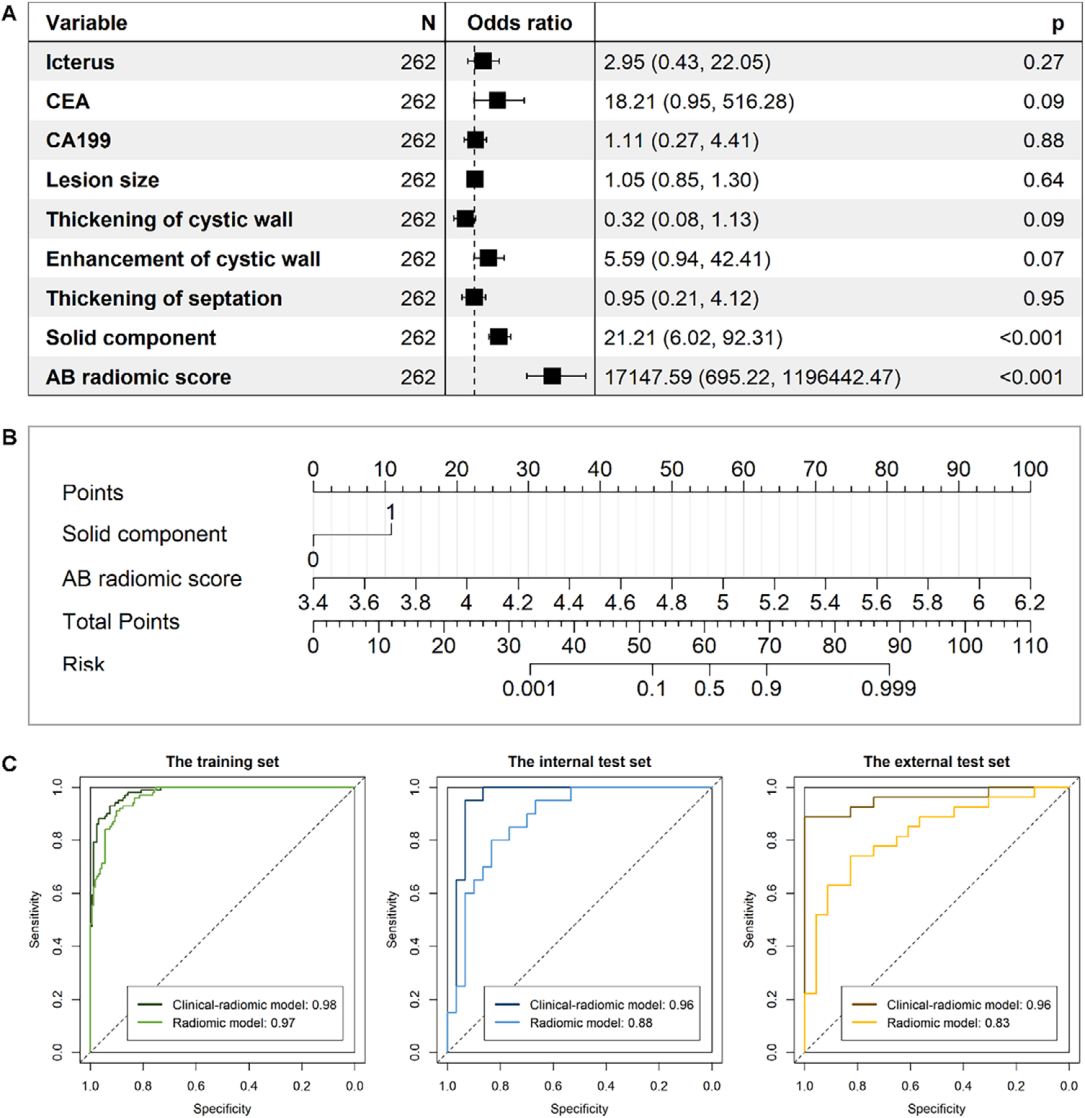
Construction and performance of the AB based clinical‐radiomic model. A) Forest plot of predictors in the training set. B) The clinical‐radiomic model presented with a nomogram scaled by the logistic regression coefficient of each predictor. C) Performance of the radiomic model and the corresponding clinical‐radiomic model with ROC analysis in the training set, internal test set, and external test set.

**Figure 6:**
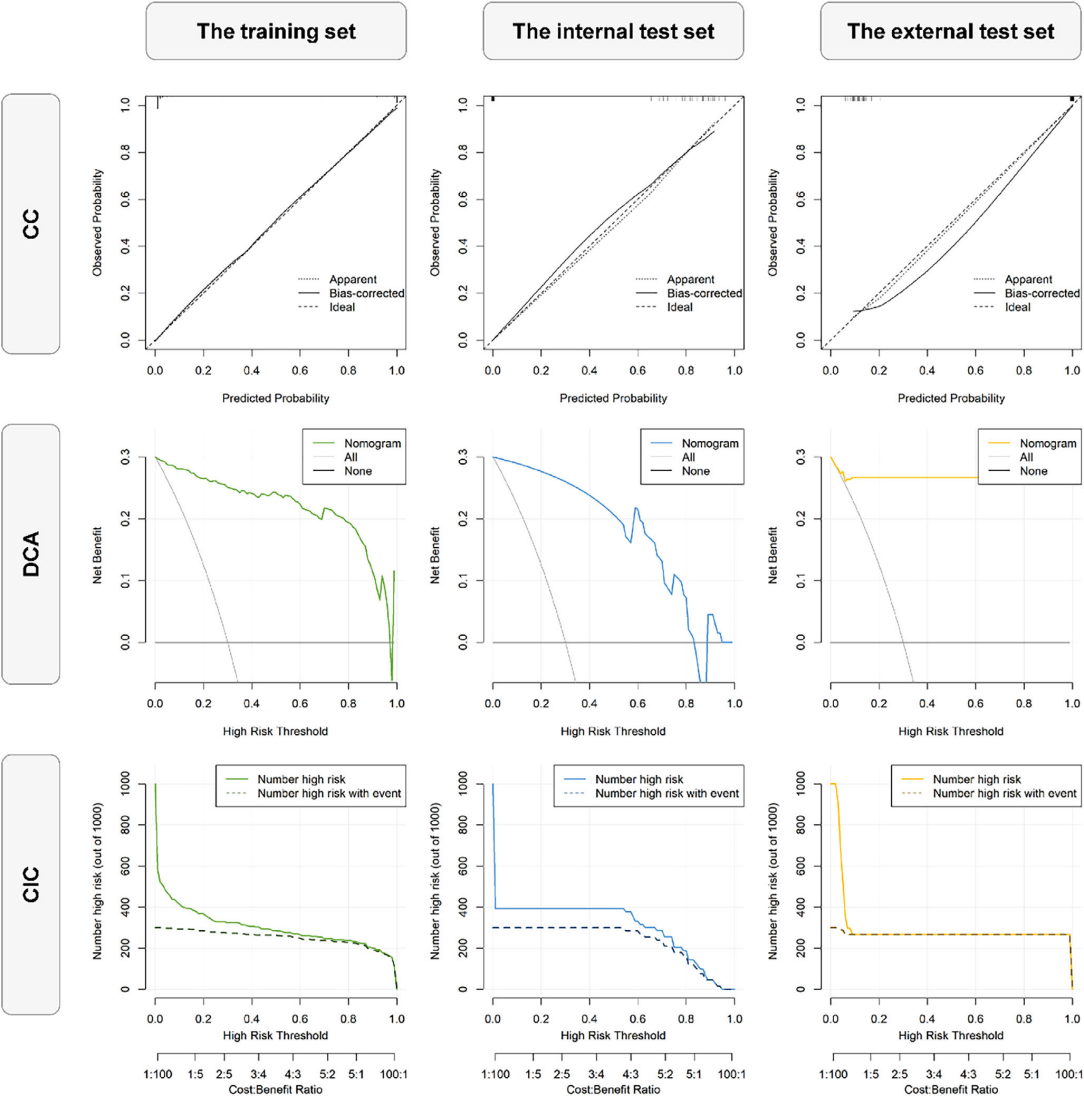
Performance analysis of AB classifier based clinical‐radiomic model for predicting benign and malignant pancreatic cystic tumors. The three rows from top to bottom represent calibration curve (CC) analysis, decision curve nalysis (DCA) analysis and clinical impact curve (CIC) analysis respectively. The three columns from left to right represent performance on the training set, the internal test set and the external test set respectively.

Taking the AB (adaptive boosting) classifier as an example, **Figures** **5** and **6** show the clinical‐radiomic nomogram model generated based on the AB radiomic score and its performance on different datasets. The figures show that the predictive performance of the AB clinical‐radiomic model (training, AUC = 0.98; internal test, AUC = 0.96; external test, AUC = 0.96) is further improved compared to the corresponding AB radiomic model (training, AUC = 0.97; internal test, AUC = 0.88; external test, AUC = 0.83).

### 2.4 Application of Generated Models in Prospective Cohort

**Figure 7:**
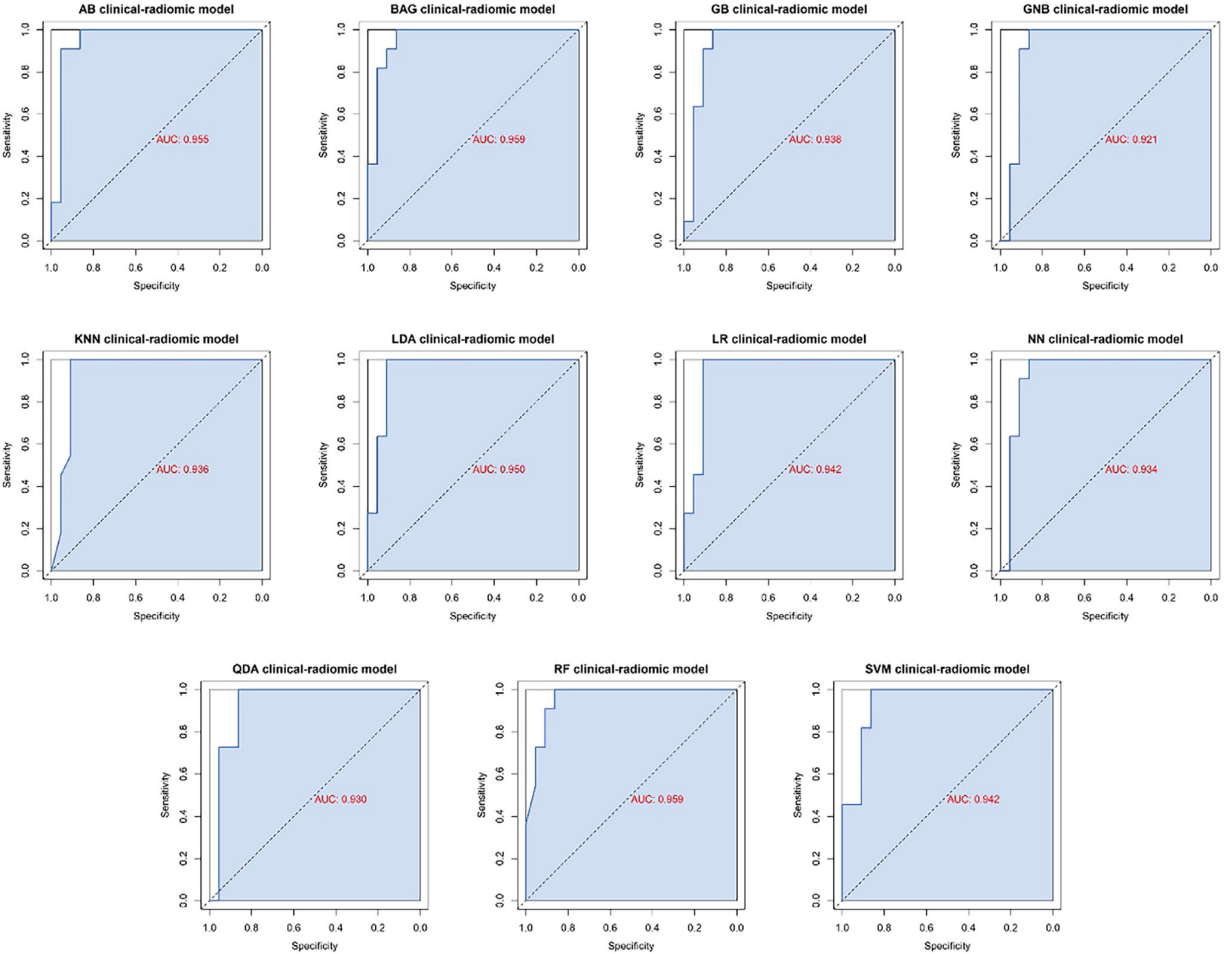
ROC curves of the clinical‐radiomic models on the prospective test set. Performance of different clinical‐radiomic models for predicting benign and malignant of pancreatic cystic neoplasm with ROC curve analysis in the prospective test set.

In our prospective cohort, 34 patients were finally included (Figure 1B), with clinical characteristics shown in Table S6 (Supporting Information). Eleven generated clinical‐radiomic machine learning models were applied in the prospective application set, with the results of AUCs ranging from 0.921 to 0.959 (**Figure** **7**).

### 2.5 Protein Expression Patterns Associated with the Clinical‐Radiomic Model

**Figure 8:**
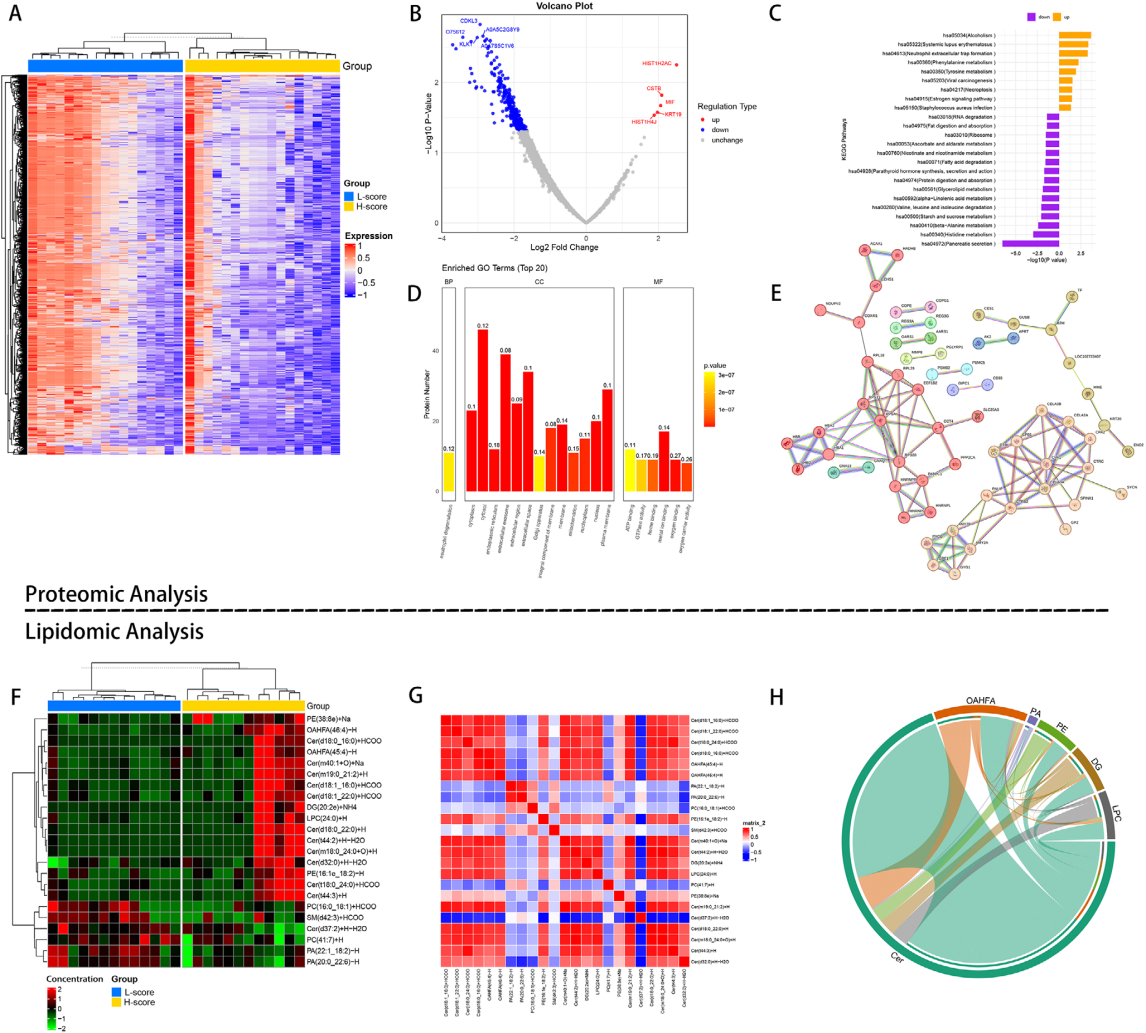
Proteomic and lipidomic analysis of protein and lipid expression patterns associated with the clinical‐radiomics model. A) Heatmap shows the expression levels of differentially expressed proteins between groups stratified by the clinical‐radiomics model score in the proteomics sample. B) Volcano plot shows the differentially expressed proteins in the low‐score group compared with the high‐score group. C) Bar plots show the biological process involved of these differentially expressed proteins according to the GO enrichment analysis. D) KEGG pathway enrichment analysis of differentially expressed proteins between low‐ and high‐score group. E) Protein‐protein interaction network of differentially expressed proteins. F) Heatmap shows the expression levels of differentially expressed lipid molecules between groups stratified by the clinical‐radiomics model score in the lipidomics sample. G) Correlation analysis shows the metabolic proximities of differentially expressed lipid molecules. H) The chord diagrams reveal the most abundant lipid class with maximum connectivity.

The differentially expressed proteins between the high and low clinical‐radiomic score groups from the prospective cohort were shown in **Figure** **8**A,B. Three hundred and forty‐seven differentially‐expressed proteins were identified among 3415 tested proteins. Interestingly, only 5 upregulated proteins were observed, including histone H2A type1 (HIST1H2A), cystatin‐B (CSTB), Macrophage migration inhibitory factor (MIF), keratin19 (KRT19), and histone H4 type1 (HIST1H4). The top 5 downregulated proteins included chymotrypsinogen B2 (CTRB2), chymotrypsin‐like elastase family member 3A (CELA3A), carboxylic ester hydrolase 1 (CES1), chymotrypsin‐C (CTRC), alpha‐amylase 2B (AMY2B). The Gene Ontology (GO) enrichment analysis revealed that these proteins mainly involved in biological process of neutrophil degranulation (Figure 8C). According to Kyoto Encyclopedia of Genes and Genomes (KEGG) pathway enrichment, the up‐regulated proteins were mainly enriched into phenylalanine metabolism, mucin type O‐glycan biosynthesis, and Th17 cell differentiation; while the down‐regulated proteins were mainly enriched into biosynthesis of unsaturated fatty acids, pancreatic secretion, and starch and sucrose metabolism (Figure 8D). The results of protein‐protein interaction analysis include 119 proteins, in which 46 proteins were predicted to have protein‐protein interactions. Most of the proteins were found to be involved in the process of pancreatic secretion and eukaryotic translation elongation (Figure 8E).

### 2.6 Lipid Expression Patterns Associated with the Clinical‐Radiomic Model

A total of 28 lipid classes including 468 lipid molecules were identified in 25 cyst fluid samples, and 33 lipid molecules were detected to be differentially expressed between the high and low clinical‐radiomic score groups (Figure 8F). Correlation analysis was performed to evaluate the metabolic proximities of these differentially expressed lipid molecules (Figure 8G). The lipid‐lipid correlation matrix was transformed to the chord diagram (Figure 8H), displaying that ceramide (Cer) was the most abundant lipid class. The top 5 lipid molecules with the highest variable importance in projection (VIP) values included Cer(d32:0)+H‐H2O↑, phosphatidylethanolamin (PE) (16:1e_18:2)‐H↑, phosphatidylcholine (PC) (16:0_18:1)+HCOO↓, phosphatidic acid (PA) (22:1_18:2)‐H↓, PA(20:0_22:6)‐H↓.

## 3 Discussion

Previously reported radiomic models for classifying PCLs have been limited by small sample sizes and a lack of biological explanation of potential radiomic features.^[^ ^11^, ^16^, ^17^, ^18^, ^19^ ^]^ In this study, we developed 11 machine learning‐based radiomic models to distinguish PCLs with high‐malignant potential from those at low risk, using retrospective and prospective cohorts. Our models showed an ideal predictive performance with AUCs > 0.93 among the training, internal test, external test, and prospective application sets. The top‐performing model, based on random forest (RF), achieved AUCs of 1.00, 0.95, 0.96, and 0.96 in the training, internal test, external test, and prospective application sets, respectively. In the nomogram of our radiomic model, a high radiomic score indicated a higher malignant potential. Unlike previous studies that focused on histological types,^[^ ^18^, ^20^, ^21^ ^]^ our approach simplified classification into high‐ and low‐malignant potential groups, which could better inform clinical decision‐making regarding surgical interventions.^[^ ^4^ ^]^ Other studies developing radiomic models for distinguishing high from low malignant potential PCLs reported AUCs ranging from 0.71 to 0.92.^[^ ^22^, ^23^ ^]^ Cyst fluid analysis presents a promising technique for assessing the malignant potential of PCLs. As reported before, cyst fluid glycoproteomic analysis showed AUCs ranging from 0.771 to 0.948, comparable to our models.^[^ ^21^ ^]^ However, unlike radiomic approach, obtaining cyst fluid via EUS‐FNA is invasive. Compared to previous research, our model demonstrated superior performance in terms of reliability and generalizability, underscoring the practical utility of radiomics in accurately predicting PCL malignancy.^[^ ^19^ ^]^ Additionally, substantial studies indicated that the combination of clinical and radiomic features in prediction models could improve their performance.^[^ ^24^, ^25^ ^]^ However, given the black‐box‐like nature of the models, its further application has been significantly hindered.^[^ ^12^ ^]^ Unlike previous studies that did not delve into the “black box” of models,^[^ ^22^, ^23^ ^]^ our study concurrently conducted preoperative analysis of pancreatic cyst fluid proteomics and lipidomics in a prospective cohort, offering insights into the biological mechanisms underlying clinical‐radiomic models.

Consistent with our study, several studies have reported increased CSTB,^[^ ^26^, ^27^ ^]^ MIF,^[^ ^28^, ^29^ ^]^ and KRT19^[^ ^30^ ^]^ levels in pancreatic cancer. The other two upregulated proteins were HIST1H2 and HIST1H4, both of which were released during cell death, including necrosis and apoptosis.^[^ ^31^ ^]^ However, the relationship between extracellular histone and tumor remained unclear. Chen, L. et.al. reported that extracellular histone promoted the progression of prostate cells,^[^ ^32^ ^]^ while Martín‐Antonio, B. et.al. indicated that natural‐killer (NK) cells could release histone to active anti‐tumor immunity.^[^ ^33^ ^]^ Our analysis of the top five downregulated proteins, KEGG enrichment, and protein‐protein interaction results revealed a strong link between pancreatic secretion and the clinical‐radiomic model. Four of five downregulated proteins, including CTRB2, CELA3A, CTRC, and AMY2A, were found to be involved in pancreatic secretion according to KEGG enrichment and protein‐protein interaction analysis. A few studies about proteomics showed downregulated CTRB2,^[^ ^34^ ^]^ CELA3A,^[^ ^34^ ^]^ and CTRC,^[^ ^34^, ^35^ ^]^ in pancreatic cancer, similar to the results of our study. Van der Waaij, L. A. et.al. indicated that cystic fluid from mucinous cystadenocarcinoma had lower amylase concentration than SCN, MCN, and IPMN.^[^ ^36^ ^]^ Since the clinical‐radiomic models showed great performance in distinguishing benign and malignant PCLs, the differentially‐expressed proteins may only correlate with the malignancy of PCLs. However, the proteins involve in pancreatic secretion could significantly influence the components in cystic fluid. Thus, we infer that the different components in cystic fluid between benign and malignant PCLs may lead to different radiomic scores. However, limited studies have explored the secretory function of PCLs. Further investigation into the secretory function of PCLs could unveil novel biomarkers and insights into their origins.

Another group of proteins involved in eukaryotic translation elongation also caught our attention. This cluster, primarily consisting of ribosomal proteins (RPL18, RPL29, RPS12, RPSAc, and RPS20), did not align well with previous studies. Li, Chaodong et.al. reported that PRL29 could help with proliferation of pancreatic cancer cells.^[^ ^37^ ^]^ Two cell experiment results indicated that RPSA served as a factor that regulate the migration of pancreatic cancer cells.^[^ ^38^, ^39^ ^]^ However, another study found reduced RPSA immunohistochemical staining in pancreatic cancer compared to peripheral pancreatic tissue.^[^ ^40^ ^]^ According the studies above, this cluster of proteins served as a regulator of pancreatic cancer cells. Thus, we propose that this cluster of proteins does not influence cystic fluid composition but instead reflects correct categorization of malignant and benign PCLs. However, the discrepancy between cell experiments and proteomic findings warrants further investigation.

In our lipidomic analysis, we identified several lipid molecules (Cer(d32:0)+H‐H2O↑, PE(16:1e_18:2)‐H↑, PC(16:0_18:1)+HCOO↓, PA(22:1_18:2)‐H↓, PA(20:0_22:6)‐H↓) that potentially contribute to explaining clinical‐radiomic models. Cer emerged as the predominant lipid class, exhibiting extensive connectivity with other lipid molecules. Higher levels of Cer and PE were consistently associated with elevated clinical‐radiomic scores. Cer serves as an intracellular signaling molecule crucial for cell growth, division, and apoptosis pathways.^[^ ^41^ ^]^ Previous lipidomic studies on pancreatic cystic tumors indicated altered lipid metabolism in high‐risk IPMNs compared to serous cystic tumors, characterized by increased fatty acids and Cer levels,^[^ ^42^ ^]^ aligning with our findings. Another study reported higher Cer levels in pancreatic cancer tumors with lymph node metastasis compared to those without metastasis.^[^ ^43^ ^]^ However, Cer was regarded as an anti‐tumor molecule inducing tumor cell apoptosis.^[^ ^44^, ^45^ ^]^ Further investigation is needed to explain this contradiction. PE, the second most abundant phospholipid in eukaryotic cell membranes, influences membrane structure, oxidative phosphorylation, mitochondrial biogenesis, membrane fusion, and autophagy.^[^ ^46^ ^]^ Consistent with our results, a lipidomic study found elevated PE levels in IPMN cyst fluid compared to SCN, underscoring its relevance in cystic tumor biology.^[^ ^42^ ^]^ PA is a key intermediate in lipid metabolism and also functions as a second messenger in various signaling pathways.^[^ ^47^ ^]^ Several phospholipases that break down PA into lysophosphatidic acid and free fatty acids have been shown to be upregulated in pancreatic cancer, promoting glycolysis, consistent with our findings.^[^ ^48^, ^49^ ^]^ However, the PC level did not align with previous studies. While one study reported a downregulation of PC in the serum of PDAC patients compared to healthy individuals,^[^ ^50^ ^]^ another study found increased PC levels in PDAC tissue compared to adjacent non‐cancerous tissue.^[^ ^51^ ^]^ Given the great contrast between lipids and cystic fluid on CT images, the differential lipids may hold significant value in the interpretation of clinical‐radiomic models due to their strong association with the model scores.

Our study had several limitations. First, Despite the large dataset in our training set, the sample size of our external and prospective application sets was limited. Larger sample size of external and prospective validation cohorts should be collected in the future. Second, the radiomic models were generated based on retrospectively gathered data from patients who underwent surgical resection for PCLs, which may introduce inevitable selection bias. Third, the algorithms for processing radiomic features are not standardized between different studies, which calls for the formulation of consensus in the area of radiomic studies. Finally, Buscaglia et al. found that pancreatic cyst malignancy was independently associated with white race,^[^ ^52^ ^]^ thus the origin or ethnicity of the patients selected for this study may also have an impact on the outcome, which calls for a global multicenter study in the future.

In conclusion, our machine‐learning based models could noninvasively predict the malignant potential of PCLs with satisfactory performance and generalizability. Notably, we uncovered the alteration of pancreatic secretion function and lipid metabolism underlying the clinical‐radiomic model, suggesting the potential of our models in guiding clinical decisions.

## 4 Experimental Section

This study included 3 retrospective data sets and 1 prospective data set from 3 independent medical centers, which was approved by all institutional review boards. The approval numbers are I‐22PJ538, 2024‐HXKT‐002, and PYRC‐2024‐171‐01. Written informed consent was waived for the retrospective data sets. All patients from the prospective cohort signed the written informed consent for permission for radiomic, proteomic, and lipidomic analysis.

### Study Patients

Patients who underwent surgical resection for PCLs in the training set and internal test set were all retrospectively recruited from institution 1 between July 2014 and August 2020 for the construction of the radiomic model. Another retrospective cohort was recruited from institution 2 between May 2012 and February 2021 for the external validation of the model. The inclusion criteria were as follows: (1) pathological confirmation of PCLs with available pathology slides; (2) contrast‐enhanced CT images within 2 weeks before surgery. The exclusion criteria were as follows: (1) receiving other treatments before surgery; (2) unqualified radiological or pathological images. Clinical indicators, including age, sex, history of icterus and pancreatitis, preoperative level of CEA and CA199 within 1 week, and location of the cyst were collected. Conventional imaging features, including lesion size, signs of IPMN, the diameter of the main pancreatic duct, thickening or enhancement of the cystic wall and septation, and the presence of septation and solid components were also recorded. Patients with suspected PCLs prospectively enrolled from institution 3 between May 2019 and October 2021 received EUS‐FNA. Pancreatic cyst fluid samples were obtained before surgery and stored at ‐80 °C. These samples were used for proteomic analysis using 4D data‐independent acquisition (DIA) on timsTOF Pro 2 platform (Bruker) and lipidomic analysis using ultra‐high performance liquid chromatography‐tandem mass spectrometry (UHPLC‐MS/MS) on Q Exactive Plus platform (Thermo Scientific). Details of patient recruitment process are shown in Figure 1.

Pathology slides were reviewed by a senior pathologist with 18 years of experience in pancreatic disease diagnosis to decide the type of PCLs. Low to intermediate‐grade dysplasia of IPMN, pseudocysts, neuroendocrine tumor (G1/G2), SCN, and MCN were defined as benign PCLs. PDAC, high‐grade dysplasia & invasive IPMN, neuroendocrine tumor (G3), and solid pseudopapillary tumor (SPT) were defined as malignant PCLs.

### Radiomic Feature Extraction

**Figure 9:**
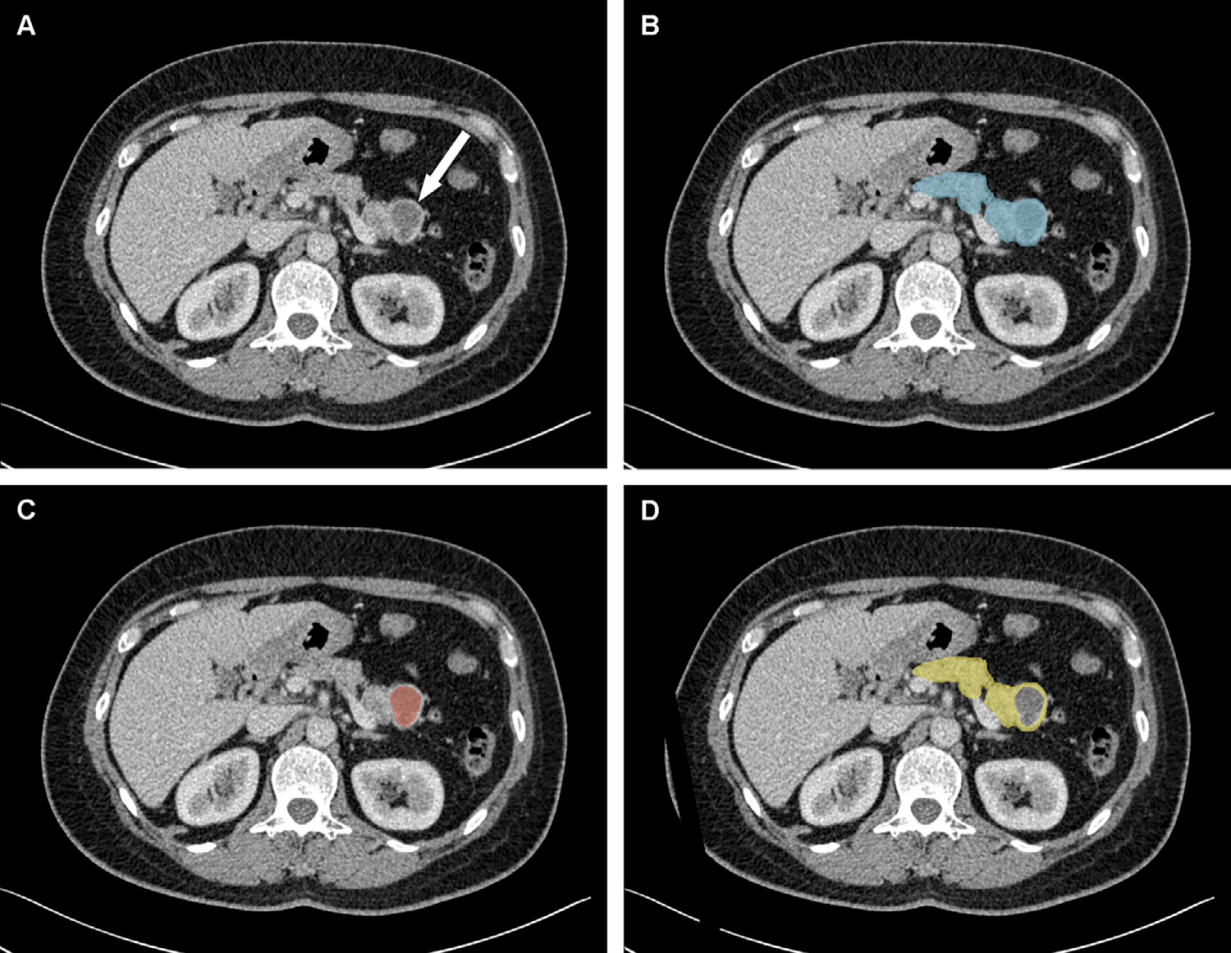
Representative segmentation results of pancreatic cystic neoplasm. A) An axial CT scan in the portal venous phase shows pancreatic cystic neoplasm (arrow). B) The pancreas segmentation mask (blue). C) The lesion segmentation mask (red). D) The non‐lesion segmentation mask (yellow) obtained by subtracting the lesion region from the pancreatic region.

A senior radiologist with 15 years of experience in abdominal disease, blinded to clinical information and pathological subtype, performed image segmentation using 3D Slicer software (version 5.6.2). The pancreas and cystic lesion were manually segmented slice by slice on portal venous phase images, and non‐lesion regions were further obtained by subtracting the lesion region from the pancreatic region (**Figure** **9**). All segmentations were confirmed by a senior radiologist and disagreements were resolved by consensus.

The radiomic features of the pancreas, lesion, and non‐lesion regions were calculated automatically using the PyRadiomics package (version 3.1.0).^[^ ^53^ ^]^ 107 features of each region were extracted, including seven types: 14 shape features, 18 first‐order features, 24 gray level co‐occurrence matrix (GLCM) features, 14 gray level dependence matrix (GLDM) features, 16 gray level run length matrix (GLRLM) features, 16 gray level size zone matrix (GLSZM) features, and 5 neighboring gray tone difference matrix (NDTGM) features. A total of 321 features were extracted for each patient for further analysis.

### Radiomic Feature Selection

The selection of radiomic features consists of three steps: standardization, univariable feature selection, and multivariable dimensionality reduction. Feature standardization is achieved by Z‐score, which aims to normalize the data to a standard normal distribution to ensure comparability between features. Univariable feature selection is to retain the features with significant differences through statistical tests (see “Statistical analysis” section). Multivariable feature selection was based on LASSO regression with five‐fold cross‐validation to produce more interpretable and non‐collinear features while achieving feature dimensionality reduction. The above processes were implemented by Python (version 3.7) programming.

### Radiomic Model Generation

Eleven common machine learning (ML) classifiers were used to develop radiomic models based on the selected features. The classifiers included adaptive boosting (AB), bagging (BAG), gradient boosting (GB), Gaussian Naive Bayes (GNB), k‐nearest neighbor (KNN), linear discriminant analysis (LDA), logistic regression (LR), neural network (NN), quadratic discriminant analysis (QDA), random forest (RF), and support vector machine (SVM).

The radiomic models were generated as follows: a) Patients from our hospital (hospital 1) were divided into a training set and an internal test set according to the chronological order of CT examination. Patients from another hospital (hospital 2) constituted the external test set. b) Construct ML models using the Scikit‐learning package (version 0.17.1) based in the final selected radiomic features on the training set. The hyperparameters were tuned using GridSearchCV function to stabilize and optimize the model performance. GridSearchCV is a method that encapsulates grid search and cross‐validation, which can automatically adjust parameters and return the best combination. c) Test and evaluate the performance of ML models on the internal and external test sets.

### Clinical‐Radiomic Model Generation

Based on the radiomic classifiers, eleven corresponding clinical‐radiomic models for the prediction of benign and malignant PCLs were also established. a) Screening of clinical characteristics by univariable analysis in the training set. b) Rescaling of the predictive probability of the radiomic model to 0–10 as the radiomic score. c) Multivariable logistic regression analysis of selected clinical features and radiomic score. d) Construct nomogram models based on features that retain statistical significance in multivariate analysis. e) Evaluation of model performance on internal and external test sets. The above processes were implemented by R software (version 4.3.2).

### Application of Generated Clinical‐Radiomic Models in the Prospective Application Set

Clinical and radiomic features were extracted and incorporated into generated clinical‐radiomic models, with ROC curves plotted and AUCs calculated to evaluate their performance in the prospective application set.

### Analysis of Proteomic and Lipidomic Results Stratified by Clinical‐Radiomic Model Scores in the Prospective Set

In the prospective application set, patients were stratified into low‐ and high‐score groups based on the median value of the clinical‐radiomic model scores (−8.98). Proteomics analysis was performed in R (version 4.1.3). A linear model was first generated by fitting the expression matrix to the design matrix using the linear model in limma (3.50.3). Next, the make Contrasts function was used to construct a contrast matrix to compare the differences between the groups. Then, the contrast matrix was applied to the linear model object to obtain a new model, which was subjected to Bayesian statistical processing to enhance model stability. Differential analysis results were extracted from the processed model object, and the Benjamini‐Hochberg method was used to correct the P‐values to obtain all results. To clearly mark differentially expressed proteins, the results were filtered and classified based on the criteria of P‐value less than 0.05 and absolute value of log fold change greater than 0.5. Hierarchical clustering algorithms were then used to classify differentially‐expressed proteins to analyze protein expression patterns within and between different groups, and the ComplexHeatmap (2.10.0) package was used to generate heatmaps to display differences within and between groups. Additionally, volcano plots were drawn using the fold change and P‐value (T‐test) of proteins in the groups as criteria. Significantly downregulated proteins were marked in blue (FC < 0.67 and *p* < 0.05), significantly upregulated proteins were marked in red (FC > 1.5 and *p* < 0.05), and non‐differentially‐expressed proteins were marked in gray, with the top 5 most significant differentially‐expressed proteins annotated. Then, Blast2Go (https://www.blast2go.com/) was used to perform GO functional annotation on all differentially‐expressed proteins. Fisher's Exact Test was then used to compare the GO functional annotation results of differentially expressed proteins in each group with all proteins identified in the experiment to determine significant differences (*p* < 0.05). The enrichment of the top 20 GO terms under all major GO categories was displayed using bar charts by ggplot2 (version 3.4.2). Blast2Go was also used to perform KEGG functional annotation on all differentially‐expressed proteins, then the differentially expressed proteins were separated into upregulated and downregulated groups, Fisher's Exact Test was used to perform pathway enrichment analysis (*p* < 0.05), which were displayed using bar charts by ggplot2 (version 3.4.2). To further explain the result of proteomics, the differentially‐expressed proteins (logFC < −2 or logFC > 2) were input into STRING (RRID:SCR_0 0 5223, version 12.0). The minimum required interaction score was set to high confidence (0.700). K‐means clustering was applied for clustering with “12” as number of clusters and “dotted line” as the edges between clusters.

Lipidomics analysis was performed in R (version 4.1.3). An OPLS‐DA model was first generated using the ropls package (version 1.26.4). Next, lipid molecules with VIP > 1 from the OPLS‐DA model and *p* < 0.05 from t‐test were used as the screening criteria for significantly differential lipid molecules. Hierarchical clustering analysis was performed on the differential lipids of the two groups using the expression levels of significant differential lipids, and the results were displayed using heatmaps generated by the ComplexHeatmap (2.10.0) package. The correlation between significantly differentially expressed lipids was calculated to obtain a correlation matrix, which was visualized as a correlation clustering heatmap using the ComplexHeatmap (2.10.0) package. To more intuitively reveal the co‐regulation relationships of lipids, the lipid correlation matrix was converted into a chord diagram, which was displayed using the circlize package (0.4.16).

### Statistical Analysis

Continuous variables in different datasets (training set, internal test set, and external test set) were analyzed using one‐way ANOVA or Kruskal‐Wallis H test. Continuous variables for patients with different pathological subtypes (benign and malignant) in the training set were analyzed using Student's t or Mann‐Whitney U test. Categorical variables were analyzed using Chi‐square or Fisher exact test. A two‐sided *P* < 0.05 was considered a significant difference, but in univariable analysis, features with *P* < 0.10 were retained for further multivariable analysis.

## Conflict of Interest

The authors declare no conflict of interest.

## Supporting information



Supporting Information

## Data Availability

The data that support the findings of this study are available on request from the corresponding author. The data are not publicly available due to privacy or ethical restrictions.
