# Supplementary material for: Machine Learning‐Based Radiomics in Malignancy Prediction of Pancreatic Cystic Lesions: Evidence from Cyst Fluid Multi‐Omics
Source: Adv Sci (Weinh). 2025 Apr 28;12(20):2409488. doi: 10.1002/advs.202409488 (PMC12120750; doi:10.1002/advs.202409488)
Supplement: Supplementary file 1 — Supporting Information [file ADVS-12-2409488-s001.docx]

SUPPLEMENTARY FIGUREs


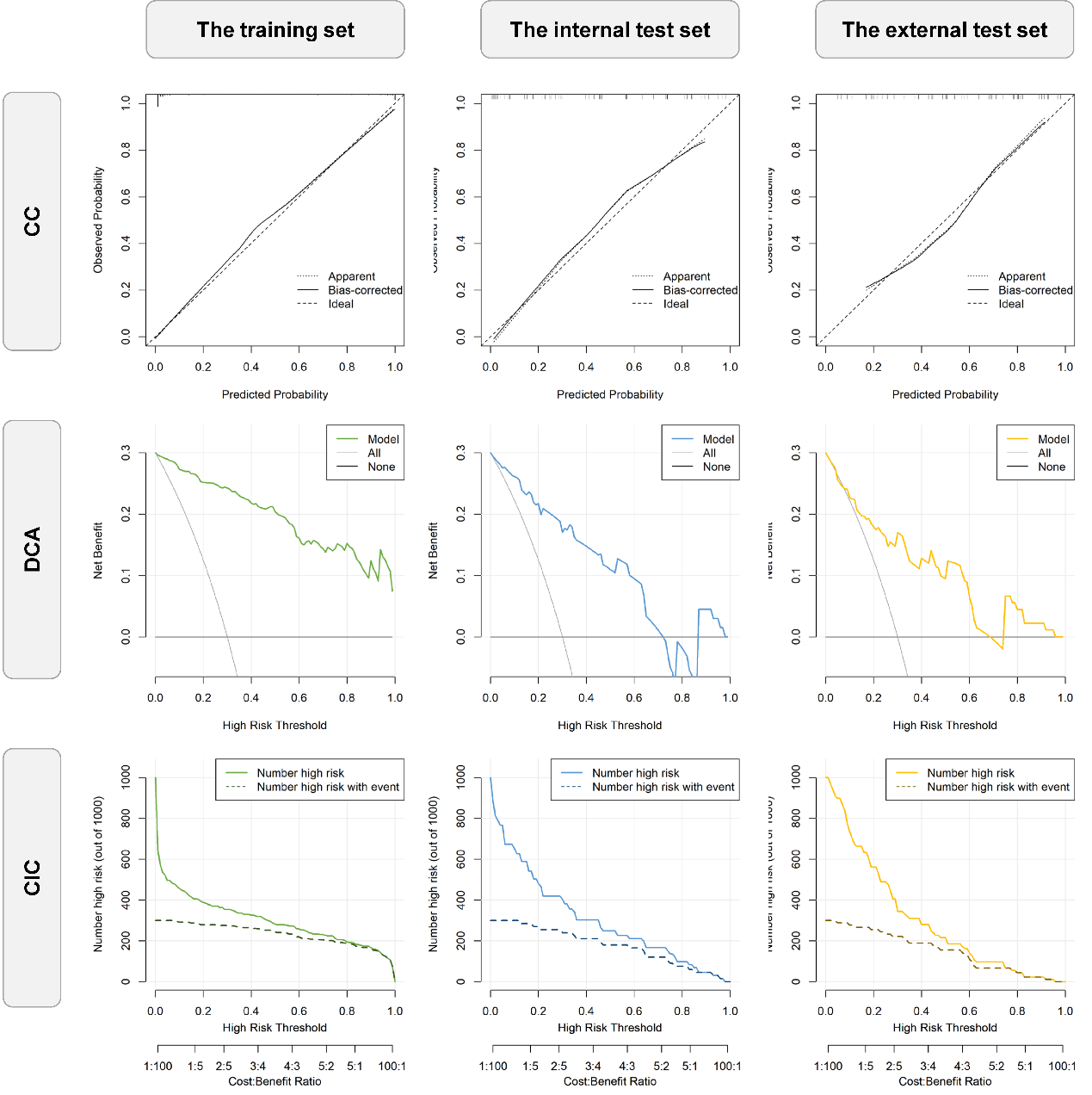


Figure S1: Performance analysis of AB classifier based radiomic model for predicting benign and malignant pancreatic cystic tumors.

The three rows from top to bottom represent CC analysis, DCA analysis and CIC analysis respectively. The three columns from left to right represent performance on the training set, the internal test set and the external test set respectively.

Abbreviations: AB = adaptive boosting, CC = calibration curve, DCA = decision curve analysis, CIC = clinical impact curve.

Tables

SUPPLEMENTARY Tables

Table S1: Clinical characteristics and visual assessment features of study patients in different datasets

| Variable | Training set (n=262) | Internal test set (n=50) | External test set (n=50) | P value |
| --- | --- | --- | --- | --- |
| ***Clinical characteristics*** |  |  |  |  |
| Age^*^ | 49 ± 15 | 51 ± 15 | 48 ± 16 | 0.754 |
| Sex (Male) | 106 (40%) | 21 (42%) | 23 (46%) | 0.764 |
| Icterus | 12 (5%) | 2 (4%) | 4 (8%) | 0.535 |
| Pancreatitis | 39 (15%) | 12 (24%) | 7 (14%) | 0.250 |
| CEA > 5 ng/ml | 24 (9%) | 5 (10%) | 3 (6%) | 0.762 |
| CA199 > 34 U/ml | 61 (23%) | 11 (22%) | 8 (16%) | 0.524 |
| Location |  |  |  | 0.050^**^ |
| Head or neck | 103 (39%) | 17 (34%) | 28 (56%) |  |
| Body or tail | 159 (61%) | 33 (66%) | 22 (44%) |  |
| Pathology |  |  |  | 0.125 |
| Benign | 161 (61%) | 30 (60%) | 23 (46%) |  |
| Malignant | 101 (39%) | 20 (40%) | 27 (54%) |  |
| ***Visual assessment features*** |  |  |  |  |
| Lesion size (cm)^*^ | 5.28 ± 2.58 | 4.53 ± 2.30 | 5.23 ± 4.70 | 0.057 |
| Communication with the main pancreatic duct | 57 (22%) | 7 (14%) | 11 (22%) | 0.450 |
| Diameter of the main pancreatic duct (mm)^*^ | 3.09 ± 2.62 | 3.57 ± 3.06 | 3.21 ± 2.64 | 0.281 |
| Dilatation of the main pancreatic duct (> 5 mm) | 53 (20%) | 13 (26%) | 12 (24%) | 0.596 |
| Thickening of cystic wall (> 2 mm) | 99 (38%) | 21 (42%) | 26 (52%) | 0.166 |
| Enhancement of cystic wall | 214 (82%) | 44 (88%) | 37 (74%) | 0.195 |
| Septation | 197 (75%) | 39 (78%) | 41 (82%) | 0.562 |
| Thickening of septation (> 2 mm) | 101 (39%) | 22 (44%) | 24 (48%) | 0.400 |
| Enhancement of septation | 194 (74%) | 39 (78%) | 40 (80%) | 0.603 |
| Solid component | 94(36%) | 24(48%) | 24(48%) | 0.107 |

Unless otherwise indicated, data are numbers with percentages in parentheses.

^*^ Data are means ± standard deviations.

^**^ The P value with more decimal places for the variable "location" is 0.050415.

*Abbreviations:* CEA = carcinoembryonic antigen, CA199 = carbohydrate antigen 19-9.

Table S2: Clinical characteristics and visual assessment features of benign and malignant study patients in different datasets

| Variables | Training set | | Internal test set | | External test set | |
| --- | --- | --- | --- | --- | --- | --- |
|  | Benign (n=161) | Malignant (n=101) | Benign (n=30) | Malignant (n=20) | Benign (n=23) | Malignant (n=27) |
| ***Clinical characteristics*** |  |  |  |  |  |  |
| Age^*^ | 50 ± 13 | 47 ± 17 | 51 ± 16 | 50 ± 14 | 54 ± 13 | 43 ± 17 |
| Sex (Male) | 64 (40%) | 42 (42%) | 11 (37%) | 10 (50%) | 11 (48%) | 12 (44%) |
| Icterus | 4 (3%) | 8 (8%) | 1 (3%) | 1 (5%) | 2 (9%) | 2 (7%) |
| Pancreatitis | 28 (17%) | 11 (11%) | 7 (23%) | 5 (25%) | 6 (26%) | 1 (4%) |
| CEA > 5 ng/ml | 6 (4%) | 18 (18%) | 2 (7%) | 3 (15%) | 1 (4%) | 2 (7%) |
| CA199 > 34 U/ml | 31 (19%) | 30 (30%) | 3 (10%) | 8 (40%) | 2 (9%) | 6 (22%) |
| Location |  |  |  |  |  |  |
| Head or neck | 63 (39%) | 40 (40%) | 8 (27%) | 9 (45%) | 14 (61%) | 14 (52%) |
| Body or tail | 98 (61%) | 61 (60%) | 22 (73%) | 11 (55%) | 9 (39%) | 13 (48%) |
| ***Visual assessment features*** |  |  |  |  |  |  |
| Lesion size (cm)^*^ | 5.13 ± 2.63 | 5.53 ± 2.50 | 4.15 ± 1.89 | 5.10 ± 2.77 | 5.05 ± 6.65 | 5.38 ± 2.05 |
| Communication with the main pancreatic duct | 34 (21%) | 23 (23%) | 5 (17%) | 2 (10%) | 7 (30%) | 4 (15%) |
| Diameter of the main pancreatic duct (mm)^*^ | 3.00 ± 2.51 | 3.25 ± 2.80 | 3.68 ± 3.43 | 3.40 ± 2.45 | 3.63 ± 2.54 | 2.84 ± 2.73 |
| Dilatation of the main pancreatic duct (> 5 mm) | 29 (18%) | 24 (24%) | 7 (23%) | 6 (30%) | 8 (35%) | 4 (15%) |
| Thickening of cystic wall (> 2 mm) | 37 (23%) | 62 (61%) | 6 (20%) | 15 (75%) | 3 (13%) | 23 (85%) |
| Enhancement of cystic wall | 118 (73%) | 96 (95%) | 24 (80%) | 20 (100%) | 12 (52%) | 25 (93%) |
| Septation | 112 (70%) | 85 (84%) | 20 (67%) | 19 (95%) | 14 (61%) | 27 (100%) |
| Thickening of septation (> 2 mm) | 37 (23%) | 64 (63%) | 9 (30%) | 13 (65%) | 2 (9%) | 22 (82%) |
| Enhancement of septation | 109 (68%) | 85 (84%) | 20 (67%) | 19 (95%) | 13 (57%) | 27 (100%) |
| Solid component | 11 (7%) | 83 (82%) | 4 (13%) | 20 (100%) | 0 (0%) | 24 (89%) |

Unless otherwise indicated, data are numbers with percentages in parentheses.

^*^ Data are means ± standard deviations.

*Abbreviations:* CEA = carcinoembryonic antigen, CA199 = carbohydrate antigen 19-9.

Table S3: Performance of the machine learning models based on radiomic features on the training set

| Model | AUC | Sensitivity | Specificity | Accuracy | PPV | NPV | F1-score | G-means |
| --- | --- | --- | --- | --- | --- | --- | --- | --- |
| AB | 0.97 [0.94, 0.99] | 91 (92/101) | 90 (145/161) | 90 (237/262) | 85 (92/108) | 94 (145/154) | 91 | 91 |
| BAG | 1.00 [1.00, 1.00] | 100 (101/101) | 100 (161/161) | 100 (262/262) | 100 (101/101) | 100 (161/161) | 100 | 100 |
| GB | 1.00 [1.00, 1.00] | 100 (101/101) | 100 (161/161) | 100 (262/262) | 100 (101/101) | 100 (161/161) | 100 | 100 |
| GNB | 0.82 [0.76, 0.87] | 75 (76/101) | 77 (124/161) | 76 (200/262) | 67 (76/113) | 83 (124/149) | 76 | 76 |
| KNN | 0.82 [0.77, 0.88] | 76 (77/101) | 73 (118/161) | 74 (195/262) | 64 (77/120) | 83 (118/142) | 75 | 75 |
| LDA | 0.86 [0.81, 0.91] | 67 (68/101) | 89 (143/161) | 81 (211/262) | 79 (68/86) | 81 (143/176) | 77 | 77 |
| LR | 0.87 [0.83, 0.92] | 69 (70/101) | 89 (144/161) | 82 (214/262) | 80 (70/87) | 82 (144/175) | 78 | 79 |
| NN | 1.00 [1.00, 1.00] | 100 (101/101) | 100 (161/161) | 100 (262/262) | 100 (101/101) | 100 (161/161) | 100 | 100 |
| QDA | 0.91 [0.87, 0.95] | 96 (97/101) | 71 (115/161) | 81 (212/262) | 68 (97/143) | 97 (115/119) | 82 | 83 |
| RF | 1.00 [1.00, 1.00] | 100 (101/101) | 100 (161/161) | 100 (262/262) | 100 (101/101) | 100 (161/161) | 100 | 100 |
| SVM | 0.93 [0.89, 0.97] | 83 (84/101) | 88 (142/161) | 86 (226/262) | 82 (84/103) | 89 (142/159) | 86 | 86 |

Data for AUC are values with 95% confidence interval in square brackets. Data for sensitivity, specificity, accuracy, PPV, and NPV are percentages with numerators/denominators in parentheses. Data for F1-score and G-means are percentages.

*Abbreviations:* AUC = area under the receiver operating characteristic curves, PPV = positive predictive value, NPV = negative predict value, AB = adaptive boosting, BAG = bagging, GB = gradient boosting, GNB = Gaussian naive Bayes, KNN = k-nearest neighbors, LDA = linear discriminant analysis, LR = logistic regression, NN = neural network, QDA = quadratic discriminant analysis, RF = random forest, SVM = support vector machine.

Table S4: Performance of the machine learning models based on radiomic features on the internal test set

| Model | AUC | Sensitivity | Specificity | Accuracy | PPV | NPV | F1-score | G-means |
| --- | --- | --- | --- | --- | --- | --- | --- | --- |
| AB | 0.88 [0.77, 0.98] | 90 (18/20) | 67 (20/30) | 76 (38/50) | 64 (18/28) | 91 (20/22) | 77 | 77 |
| BAG | 0.86 [0.75, 0.98] | 65 (13/20) | 87 (26/30) | 78 (39/50) | 76 (13/17) | 79 (26/33) | 74 | 75 |
| GB | 0.84 [0.73, 0.96] | 70 (14/20) | 83 (25/30) | 78 (39/50) | 74 (14/19) | 81 (25/31) | 76 | 76 |
| GNB | 0.84 [0.72, 0.96] | 90 (18/20) | 67 (20/30) | 76 (38/50) | 64 (18/28) | 91 (20/22) | 77 | 77 |
| KNN | 0.88 [0.78, 0.99] | 95 (19/20) | 70 (21/30) | 80 (40/50) | 68 (19/28) | 95 (21/22) | 81 | 82 |
| LDA | 0.86 [0.75, 0.98] | 85 (17/20) | 67 (20/30) | 74 (37/50) | 63 (17/27) | 87 (20/23) | 75 | 75 |
| LR | 0.85 [0.73, 0.96] | 85 (17/20) | 63 (19/30) | 72 (36/50) | 61 (17/28) | 86 (19/22) | 73 | 73 |
| NN | 0.86 [0.74, 0.97] | 95 (19/20) | 77 (23/30) | 84 (42/50) | 73 (19/26) | 96 (23/24) | 85 | 85 |
| QDA | 0.87 [0.76, 0.98] | 95 (19/20) | 57 (17/30) | 72 (36/50) | 59 (19/32) | 94 (17/18) | 71 | 73 |
| RF | 0.89 [0.78, 0.99] | 65 (13/20) | 87 (26/30) | 78 (39/50) | 76 (13/17) | 79 (26/33) | 74 | 75 |
| SVM | 0.90 [0.80, 1.00] | 95 (19/20) | 60 (18/30) | 74 (37/50) | 61 (19/31) | 95 (18/19) | 74 | 75 |

Data for AUC are values with 95% confidence interval in square brackets. Data for sensitivity, specificity, accuracy, PPV, and NPV are percentages with numerators/denominators in parentheses. Data for F1-score and G-means are percentages.

*Abbreviations:* AUC = area under the receiver operating characteristic curves, PPV = positive predictive value, NPV = negative predict value, AB = adaptive boosting, BAG = bagging, GB = gradient boosting, GNB = Gaussian naive Bayes, KNN = k-nearest neighbors, LDA = linear discriminant analysis, LR = logistic regression, NN = neural network, QDA = quadratic discriminant analysis, RF = random forest, SVM = support vector machine.

Table S5: Performance of the machine learning models based on radiomic features on the external test set

| Model | AUC | Sensitivity | Specificity | Accuracy | PPV | NPV | F1-score | G-means |
| --- | --- | --- | --- | --- | --- | --- | --- | --- |
| AB | 0.83 [0.71, 0.94] | 74 (20/27) | 83 (19/23) | 78 (39/50) | 83 (20/24) | 73 (19/26) | 78 | 78 |
| BAG | 0.88 [0.78, 0.97] | 56 (15/27) | 96 (22/23) | 74 (37/50) | 94 (15/16) | 65 (22/34) | 70 | 73 |
| GB | 0.81 [0.68, 0.93] | 56 (15/27) | 91 (21/23) | 72 (36/50) | 88 (15/17) | 64 (21/33) | 69 | 71 |
| GNB | 0.85 [0.74, 0.96] | 89 (24/27) | 78 (18/23) | 84 (42/50) | 83 (24/29) | 86 (18/21) | 83 | 83 |
| KNN | 0.85 [0.74, 0.96] | 85 (23/27) | 70 (16/23) | 78 (39/50) | 77 (23/30) | 80 (16/20) | 77 | 77 |
| LDA | 0.88 [0.78, 0.97] | 70 (19/27) | 87 (20/23) | 78 (39/50) | 86 (19/22) | 71 (20/28) | 78 | 78 |
| LR | 0.87 [0.77, 0.97] | 74 (20/27) | 83 (19/23) | 78 (39/50) | 83 (20/24) | 73 (19/26) | 78 | 78 |
| NN | 0.87 [0.78, 0.97] | 59 (16/27) | 91 (21/23) | 74 (37/50) | 89 (16/18) | 66 (21/32) | 72 | 74 |
| QDA | 0.84 [0.73, 0.95] | 100 (27/27) | 52 (12/23) | 78 (39/50) | 71 (27/38) | 100 (12/12) | 69 | 72 |
| RF | 0.90 [0.81, 0.99] | 63 (17/27) | 100 (23/23) | 80 (40/50) | 100 (17/17) | 70 (23/33) | 77 | 79 |
| SVM | 0.85 [0.75, 0.96] | 81 (22/27) | 74 (17/23) | 78 (39/50) | 79 (22/28) | 77 (17/22) | 78 | 78 |

Data for AUC are values with 95% confidence interval in square brackets. Data for sensitivity, specificity, accuracy, PPV, and NPV are percentages with numerators/denominators in parentheses. Data for F1-score and G-means are percentages.

*Abbreviations:* AUC = area under the receiver operating characteristic curves, PPV = positive predictive value, NPV = negative predict value, AB = adaptive boosting, BAG = bagging, GB = gradient boosting, GNB = Gaussian naive Bayes, KNN = k-nearest neighbors, LDA = linear discriminant analysis, LR = logistic regression, NN = neural network, QDA = quadratic discriminant analysis, RF = random forest, SVM = support vector machine.

**Table S6: Clinical characteristics and visual assessment features of study patients in prospective test set**

| Characteristics | All (n=34) | Benign (n=23) | Malignant (n=11) |
| --- | --- | --- | --- |
| ***Clinical characteristics*** |  |  |  |
| Age^*^ | 59 ± 16 | 55 ± 17 | 68 ± 9 |
| Sex (Male) | 21 (62%) | 13 (57%) | 8 (73%) |
| Icterus | 1 (3%) | 0 (0%) | 1 (9%) |
| Pancreatitis | 4 (12%) | 2 (9%) | 2 (18%) |
| CEA > 5 ng/ml | 6 (18%) | 3 (13%) | 3 (27%) |
| CA199 > 34 U/ml | 9 (26%) | 2 (9%) | 7 (64%) |
| Location |  |  |  |
| Head or neck | 19 (56%) | 13 (57%) | 6 (55%) |
| Body or tail | 15 (44%) | 10 (43%) | 5 (45%) |
| ***Visual assessment features*** |  |  |  |
| Lesion size (cm)^*^ | 3.93 ± 1.88 | 3.84 ± 1.98 | 4.12 ± 1.84 |
| Communication with pancreatic duct | 13 (38%) | 9 (39%) | 4 (36%) |
| Diameter of the main pancreatic duct (mm)^*^ | 5.35 ± 4.76 | 3.62 ± 2.34 | 8.83 ± 6.59 |
| Dilatation of the main pancreatic duct > 5 mm | 13 (38%) | 5 (22%) | 8 (73%) |
| Thickening of cystic wall > 2 mm | 14 (41%) | 6 (26%) | 8 (73%) |
| Enhancement of cystic wall | 20 (59%) | 10 (43%) | 10 (91%) |
| Septation | 11 (32%) | 7 (30%) | 4 (36%) |
| Thickening of septation > 2 mm | 2 (6%) | 1 (4%) | 1 (9%) |
| Enhancement of septation | 11 (32%) | 7 (30%) | 4 (36%) |
| Solid component | 14 (41%) | 3 (13%) | 11 (100%) |

Unless otherwise indicated, data are numbers with percentages in parentheses.

^*^ Data are means ± standard deviations.

*Abbreviations:* CEA = carcinoembryonic antigen, CA199 = carbohydrate antigen 19-9.
